# The Impact of COVID-19 on Pediatric Cardiac Arrest Outcomes: A Systematic Review and Meta-Analysis

**DOI:** 10.3390/ijerph20021104

**Published:** 2023-01-08

**Authors:** Alla Navolokina, Jacek Smereka, Bernd W. Böttiger, Michal Pruc, Raúl Juárez-Vela, Mansur Rahnama-Hezavah, Zubaid Rafique, Frank W. Peacock, Kamil Safiejko, Lukasz Szarpak

**Affiliations:** 1 European School of Medicine, International European University, 03187 Kyiv, Ukraine; 2 Department of Emergency Medical Service, Wroclaw Medical University, 51-616 Wroclaw, Poland; 3 Research Unit, Polish Society of Disaster Medicine, 05-806 Warsaw, Poland; 4 Department of Anaesthesiology and Intensive Care Medicine, University Hospital of Cologne, 50937 Cologne, Germany; 5 GRUPAC, Department in Nursing, University of La Rioja, 26006 Logroño, Spain; 6 Chair and Department of Oral Surgery, Medical University of Lublin, 20-093 Lublin, Poland; 7 Henry JN Taub Department of Emergency Medicine, Baylor College of Medicine, Houston, TX 77030, USA; 8 Research Unit, Maria Sklodowska-Curie Bialystok Oncology Center, 15-294 Bialystok, Poland

**Keywords:** cardiac arrest, COVID-19, SARS-CoV-2, cardiopulmonary resuscitation, pediatric cardiac arrest, meta-analysis

## Abstract

Severe acute respiratory syndrome coronavirus 2 (SARS-CoV-2) caused a global pandemic, required the donning of personal protective equipment during clinical contact, and continues to be a significant worldwide public health concern. Pediatric cardiac arrest is a rare but critical condition with a high mortality rate, the outcomes of which may be negatively affected by donning personal protective equipment. The aim of this study is to perform a systematic review and meta-analysis of the impact of the COVID-19 pandemic on pediatric cardiac arrest outcomes. We conducted a systematic review with meta-analysis in the following databases: PubMed, EMBASE, Scopus, Web of Science, and Cochrane Library from their inception to 1 October 2022. We included studies published in English on pediatric patients with cardiac arrest, dichotomized by the pre- and during-COVID-19 periods and then stratified by COVID-19 positive or negative status, to evaluate clinical outcomes associated with cardiac arrest. Six studies were included in the meta-analysis. In witnessed out-of-hospital cardiac arrest patients, there were no differences between the pandemic and pre-pandemic periods for witnessed cardiac arrest (28.5% vs. 28.7%; odds ratio (OR) = 0.99; 95% confidence interval (CI): 0.87 to 1.14; *p* = 0.93), administration of bystander cardiopulmonary resuscitation (61.5 vs. 63.6%; OR = 1.11; 95%CI: 0.98 to 1.26; *p* = 0.11), bystander automated external defibrillator use (both 2.8%; OR = 1.00; 95%CI: 0.69 to 1.45; *p* = 0.99), return of spontaneous circulation(8.4 vs. 8.9%; OR = 0.93; 95%CI: 0.47 to 1.88; *p* = 0.85), survival to hospital admission (9.0 vs. 10.2%, OR = 0.81; 95%CI: 0.45 to 1.44; *p* = 0.47), or survival to hospital discharge (13.4 vs. 12.4%; OR = 0.62; 95%CI: 0.22 to 1.72; *p* = 0.35). COVID-19 did not change pediatric cardiac arrest bystander interventions or outcomes.

## 1. Introduction

Severe acute respiratory syndrome coronavirus 2 (SARS-CoV-2) caused a global pandemic and continues to be a significant worldwide public health concern [1,2,3]. The World Health Organization declared the outbreak a pandemic in March 2020 [4,5]. Since then, more than 600 million cases of COVID-19 have been reported, with 14% of patients requiring hospitalization and 2% requiring intensive care unit (ICU) admission [6] resulting in more than 6 million deaths [7]. This sheer volume of patients has caused tremendous stress on the global healthcare system, with concerns that critically ill patients were not being resuscitated adequately or not receiving comprehensive post-resuscitative care [8,9,10].

Pediatric cardiac arrest is a rare but critical condition with a high mortality [11,12]. Its major causes stem from respiratory failure (e.g., drowning or airway obstruction) and trauma. Out-of-hospital cardiac arrests (OHCA) in both adults and pediatrics have low survival rates [13,14], with prior studies showing that bystander cardiopulmonary resuscitation (CPR) and rapid transportation to the hospital can drastically improve outcomes [15,16]. However, with an ongoing pandemic of a highly contagious virus which requires donning personal protective equipment before caring for patient’s, bystander CPR, rapid initiation of resuscitation, and overall CPR quality may have suffered, thus affecting the overall outcome [17,18,19,20].

Several studies have evaluated pediatric cardiac arrest outcomes [12,21,22,23,24,25] with mixed results. Generally, the studies are small, since children have lower rates of critical illness and mortality in general, and specifically, from COVID-19 [26,27]. The aim of this study is to perform a systematic review and meta-analysis of the impact of the COVID-19 pandemic on pediatric cardiac arrest outcomes.

## 2. Materials and Methods

The study was designed as a systematic review and meta-analysis and was performed in accordance with the Preferred Reporting Items for Systematic Reviews and Meta-analyses (PRISMA) guidelines [28]. Institutional review board approval was not required as this study did not include individual patient data.

### 2.1. Literature Search

Two independent reviewers (A.N. and M.P.) retrieved literature from PubMed, EMBASE, Scopus, Web of Science, and Cochrane Library from their inception to October 1st, 2022. The search items mainly comprised “pediatric*” OR “paediatric*” OR “child*” AND “cardiac arrest” OR “resuscitation” OR “cardiopulmonary resuscitation” OR “CPR” OR “ROSC” OR “return of spontaneous circulation” AND “COVID-19” OR “novel coronavirus” OR “SARS-CoV-2”. The reference lists of included studies were searched manually for any potentially eligible articles. Two reviewers screened potential eligible papers independently, with any disagreement solved by further discussion or arbitrated by a third reviewer.

### 2.2. Study Selection

Inclusion criteria were as follows: (1) participants enrolled in the study were pediatric patients with cardiac arrest; (2) studies comparing cardiac arrest outcomes in the pre- and during COVID-19 periods or during COVID-19 period among patients stratified by COVID-19 status (i.e., either positive vs. negative); (3) studies evaluating the clinical outcomes of cardiac arrest (randomized and non-randomized trials); (4) studies with accessible and essential data; (5) studies published in English. The studies were excluded if they met any of the following criteria: (1) review, conference abstract, adult patients, animal experiment, case report or case series, or comment; (2) the article was not written in English; (3) basic data could not be extracted.

### 2.3. Data Extraction and Quality Assessment

Two reviewers (A.N. and R.J.-V.), using a pre-defined standardized data form, extracted data from individual manuscripts. Any discrepancies in the extracted data were identified and resolved via discussion with a third reviewer (L.S.).

The primary outcome was the incidence of return of spontaneous circulation. Secondary outcomes included the incidence of survival to hospital admission, survival to hospital discharge (SHD) and SHD with good neurological outcome defined as a cerebral performance category (CPC) 1–2.

The same reviewers evaluated the quality assessment of all included studies. We used the Newcastle–Ottawa Quality Assessment Scale (NOS) to assess the quality of each study with three aspects: the selection of patients, comparability of groups, and assessment of outcome [29]. We graded the quality of included studies as “good” (6–9) or “bad” (0–5).

### 2.4. Statistical Analysis

We conducted the meta-analysis with STATA (ver. 14.0; Stata Corporation, College Station, TX, USA) and Review Manager (ver. 5.4; The Cochrane Collaboration, Software Update, Oxford, UK) software. A two-tailed *p*-value of less than 0.05 was defined as statistically significant. The results are presented as forest plots using odds ratios (ORs) for dichotomous data and the mean difference (MD) for continuous data, with 95% confidence intervals (CIs). In case when data were reported as median with interquartile range, estimated means and standard deviations, with the formula described by Hozo, were used [30]. Heterogeneity between studies was assessed by the I^2^ test and was assessed as low, moderate, or high when I^2^ was <50%, 50–75%, or ≥76%, respectively [31]. The random-effects model was used for I^2^ > 50%; otherwise, the fixed effects model was employed. Egger’s test and funnel plots were used to assess potential bias and perform funnel plot tests for asymmetry to investigate potential publication bias if there were more than ten trials in a single meta-analysis. We perform funnel plot tests for asymmetry to investigate potential publication bias if there were more than 10 trials in a single meta-analysis.

## 3. Results

### 3.1. Search Results and Study Characteristics

**Figure 1:**
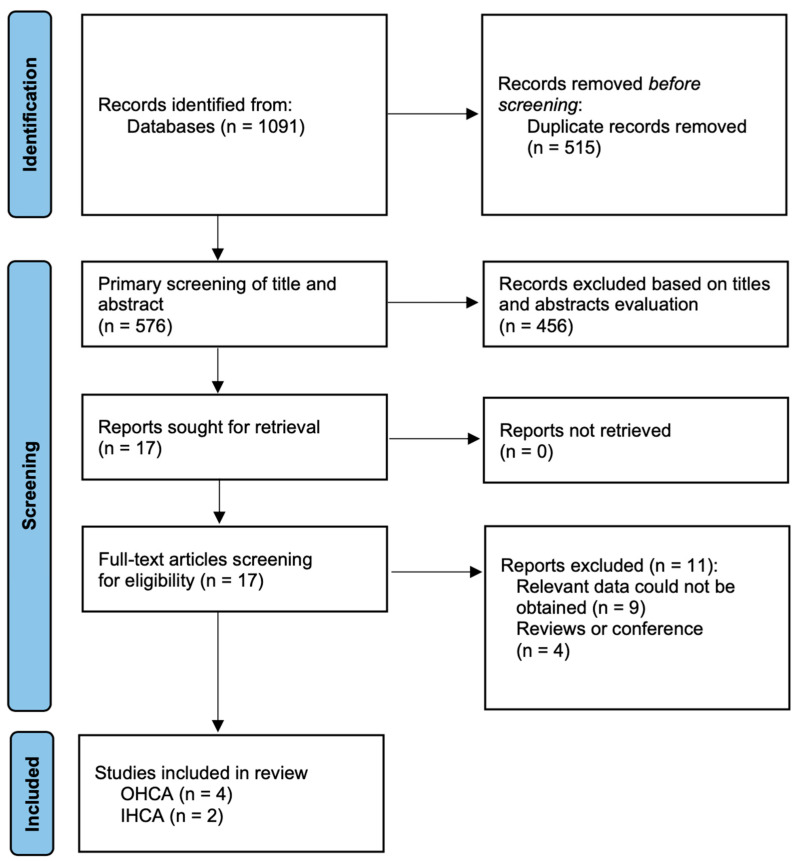
Flow diagram of the search strategy and study selection. Legend: IHCA: in-hospital cardiac arrest; OHCA: out-of-hospital cardiac arrest.

Our electronic literature search yielded 1091 potentially relevant articles and one article was identified by hand searching. After removing 515 duplicates, the remaining studies were screened for eligibility. After exclusions based on title and abstract (n = 454), we screened 17 full-text studies, and 6 studies [12,21,22,23,24,25] were finally included in the analysis (4 studies reported OHCA [12,21,24,25] and 2 studies reported in-hospital cardiac arrest (IHCA) [22,23]). Figure 1 illustrates a flow diagram describing the article selection process, which was based on the PRISMA statement.

The baseline characteristics described in Table 1 comprise a country, article type, the quality assessment score for each study, and the patients’ characteristics. The trials were published between 2020 and 2022, with sample sizes ranging from 47 to 7603 participants. All trials in OHCA were single-country and were conducted in Taiwan [21], Poland [12], France [24] and Korea [25]. In turn, both studies on IHCA were conducted in the USA [22,23]. The methodologic quality of the included trials was low and was summarized in Table 1.

### 3.2. Out-of-Hospital Cardiac Arrest

Pooled analysis showed no differences between the pre-and pandemic periods in witnessed cardiac arrest (28.5 vs. 28.7%; OR = 0.99; 95%CI: 0.87 to 1.14; *p* = 0.93; Table 2), bystander CPR (61.5 vs. 63.6%; OR = 1.11; 95%CI: 0.98 to 1.26; *p* = 0.11) or use of automated external defibrillator (AED) bystander (both 2.8%; OR = 1.00; 95%CI: 0.69 to 1.45; *p* = 0.99).

Return of spontaneous circulation was obtained at similar rates pre and during COVID. 8.4 and 8.9%, respectively (OR = 0.93; 95%CI: 0.47 to 1.88; *p* = 0.85), as occurred with survival to hospital admission, which was 9.0% vs. 10.2%, respectively (OR = 0.81; 95%CI: 0.45 to 1.44; *p* = 0.47). Survival to hospital discharge, reported in two trials, was 13.4 for the per-COVID-19 period and 12.4% during the pandemic (OR = 0.62; 95%CI: 0.22 to 1.72; *p* = 0.35). Only one study reported SHD with the good neurological outcome (CPC 1 or 2) and was 5.1% both before and during the pandemic the pandemic periods (OR = 0.99; 95%CI: 0.74 to 1.31; *p* = 0.94.

### 3.3. In-Hospital Cardiac Arrest

Only one study, published by Morgan et al., reported pediatric cardiopulmonary resuscitation outcomes during the pre and COVID-19 pandemic periods [23]. They found that shockable rhythms were observed in 6.4 vs. 9.2% of cardiac arrests before and during the pandemic period, respectively (OR = 0.67; 95%CI: 0.33 to 1.37; *p* = 0.28). In turn, bradycardia with poor perfusion was observed in 51.3 vs. 42.1%% of cases. Return of spontaneous circulation was observed among 72.2% of the pre-pandemic cases, and in 67.7% during the pandemic period (OR = 1.24; 95%CI: 0.82 to 1.88; *p* = 0.31). Survival to hospital discharge before and during the COVID-19 period was 62.4% vs. 57.9%, respectively (OR = 1.20; 95%CI: 0.82 to 1.78; *p* = 0.35). Finally, SHD, with favorable neurologic outcome was 59.8% vs. 52.3%, for pre and during the pandemic, respectively (OR = 1.36; 95%CI: 0.93 to 1.99; *p* = 0.12).

In turn, El-Zein et al. [22] reported pediatric cardiopulmonary resuscitation outcomes during the COVID-19 pandemic among positive vs. negative COVID-19 patients [22]. Pooled analysis showed that ROSC in COVID-19-positive patients was at 67.4% compared to 76.9% for negative patients (OR = 0.62; 95%CI: 0.33 to 1.16; *p* = 0.14), and SHD was 39.1% vs. 44.9%, respectively (OR = 0.79; 9%%CI: 0.43 to 1.44; *p* = 0.44).

## 4. Discussion

This is the world’s first meta-analysis on the pediatric cardiac arrest during the COVID-19 pandemic, collecting all existing sources and systematizing knowledge about this issue. The COVID-19 pandemic may have affected many elements related to the cause of cardiac arrest in pediatric patients. These includes both the patient’s underlying health status, and the quality of the procedures undertaken at each stage. In each patient’s resuscitation, the outcome is influenced by many elements, including those related to the cause of sudden cardiac arrest, the patient’s health status, comorbidities, the starting of resuscitation by lay rescuers, the use of AEDs, meeting the requirements for high-quality cardiopulmonary resuscitation, how advanced resuscitation is performed, and the quality of post-resuscitation care. Of primary importance is the issue of the organization of prehospital care, the level of care, and issues related to the timing and the quality of the procedures implemented [32].

Recurring concerns in several papers regarding the quality of response measures taken at the scene, during transport to the hospital, and at the hospital stage during the COVID-19 pandemic have been related to the safety of medical personnel and event bystanders [33,34,35]. Several recommendations by international institutions have emphasized the safety issues of providing medical care and first aid to patients with suspected or confirmed SARS-CoV-2 infection, including shortening or not undertaking resuscitation efforts in selected groups of patients where the effectiveness of the measures taken was questionable [32].

However, for social and ethical reasons, these concerns may have been of less effect in cases of assisting children, which may have the consequence of different outcomes regarding survival rates of sudden cardiac arrest compared to the adult population [36].

We performed an analysis and comparison of sudden cardiac arrest in the pediatric population before and during the COVID-19 pandemic. The studies we included aimed to evaluate for differences and identify parameters that were significantly different in the period before and during the pandemic. It should be noted, because of logistical and operational issues, most studies did not analyze the direct effect of SARS-CoV-2 infection on sudden cardiac arrest status because of the challenges of determining SARS-CoV-2 infection status of a child who has suffered sudden cardiac arrest during the pandemic.

Overall, the studies we evaluated varied and presented different groups of pediatric patients, from varying populations, in whom resuscitation efforts were undertaken at both the prehospital and in-hospital stages. The authors of the included studies considered several parameters related to cardiac arrest in the pediatric patient population, as well as the conduct of resuscitation, and its effectiveness. Differences were found in the populations analyzed, including the data acquisition method, study eligibility, and exclusion from the study.

In our meta-analysis, the primary outcome was the incidence of return of spontaneous circulation. This was selected due to the unambiguity of the defined entry criteria, the feasibility of the analysis, and the fact that this parameter has been analyzed in many resuscitation studies such that normative comparison is possible. The secondary outcomes included the incidence of survival to hospital admission, survival to hospital discharge (SHD), and SHD with good neurological outcome defined as cerebral performance categories (CPC) 1–2. This latter analysis was chosen because neurological functioning is of greater practical importance and clinical relevance after the return of spontaneous circulation as it considers the ability to return to previous social and professional roles later in life.

Regarding the out-of-hospital cardiac arrest, our analysis found no differences between the pre and during pandemic periods in witnessed cardiac arrest, bystander CPR, or use of AED bystander. The lack of differences in witnessed cardiac arrest may have been influenced by the issue of adults providing care, their presence with the child, or the presence of the child in a group that was cared for by caregivers and professionals. For ethical reasons, resuscitation in children is undertaken more often than in adults, which appears to be true regardless of the threat of SARS-CoV-2 infection or the limitations associated with the pandemic. It is important to note that using AEDs can give the rescuer a strong feeling of performing high-quality CPR without significantly increasing the risk of infection during AED use. Training the public in the general population on the use of AEDs is yielding better and better results, and these devices are being used regardless of the pandemic.

Furthermore, the arrival of the pandemic had no effect of prehospital cardiac arrest outcomes. We found that return of spontaneous circulation was obtained in only 8.4% pre-pandemic children and was 8.9% during the COVID-19 period. With such few patients arriving to the hospital alive, it is not surprising that there were no significant differences in survival to hospital admission when comparing the pre- and to the COVID-19 periods (9.0 vs. 10.2%), which was further reflected in no statistical differences in survival to hospital discharge (13.4 vs. 12.4%).

In the Morgan et al. study [23] the authors have shown that pediatric IHCA during the first year of the COVID-19 pandemic was associated with a more significant worsening of functional status and higher odds of new functional morbidity among survivors. The analysis of in-hospital cardiac arrest showed no significant differences in the survival of pediatric patients in the pre-COVID-19 period and during the pandemic. However, it should be noted that this study has a severe limitation related to the analysis of only pediatric patients who had chest compression resuscitation at the time of admission to one of the 18 ICUs. This assumption significantly affects the selection of patients and the possibility of generalizing the results.

### Limitations

As a retrospective data evaluation, our data should be considered limited to only use for hypothesis generation. Our findings cannot be considered causal in the determination of outcomes, and any specific outcomes should undergo prospective validation. After this, the main limitation of this analysis is the small size of the study groups, and that while some of the papers were published as full original papers, research letters to the editor were included. Finally, the papers included in this analysis did not always present uniform nor standardized parameters in terms of neurological outcomes in patients after out-of-hospital cardiac arrest.

## 5. Conclusions

Ultimately, the COVID-19 pandemic did not affect pediatric cardiac arrest outcomes in terms of return of spontaneous circulation, survival to hospital admission, and survival to hospital discharge.

## Figures and Tables

**Table 1:** Characteristics of included trials.

Study	Country	Cardiac Arrest Setting	COVID-19 Period			Pre-COVID-19 Period			NOS Score
			No.	Age, Years	Sex, Male	No.	AGE, YEARS	Sex, Male	
Chen et al., 2022 [21]	Taiwan	OHCA	37	NS	23 (62.2%)	60	NS	45 (75.0%)	8
Meyer-Szary et al., 2021 [12]	Poland	OHCA	29	6.7 (6.2)	15 (57.1%)	18	5.1 (6.5)	13 (72.2%)	8
Recher et al., 2020 [24]	France	OHCA	32	6 (0–14)	19 (59.4%)	53	9 (1–15)	32 (68.1%)	8
Zha et al., 2022 [25]	Japan	OHCA	1160	NS	671 (57.8%)	6443	NS	3896 (60.5%)	9
El-Zein et al., 2020 [22]	USA	IHCA	46	NS	23 (50.0%)	1282	NS	713 (55.6%)	9
Morgan et al., 2022 [23]	USA	IHCA	195	NS	93 (47.7%)	234	NS	133 (56.8%)	8

Legend: NS: not specified; IHCA: In-hospital cardiac arrest; NS: not specified; OHCA: out-of-hospital cardiac arrest.

**Table 2:** Pooled analysis of cardiac arrest characteristics.

Characteristic	No. of Studies	Event/Participants or Mean ± SD		Events		Heterogeneity between Trials		*p*-Value for Differences across Groups
		COVID-19 Period	Pre-COVID-19 Period	OR or MD	95% CI	*p*-Value	I^2^ Statistics	
Age	3	6.6 (5.5)	6.1 (5.4)	0.43	−2.73 to 3.58	0.008	79%	0.79
Sex, male	4	728/1258 (57.9%)	3988/6574 (60.7%)	0.88	0.78 to 1.00	0.46	0%	0.04
OHCA at home	1	27/31 87.1%)	32/47 (68.1%)	3.16	0.94 to 10.68	NA	NA	0.06
Cause of OHCA								
Medical	3	465/1221 (38.1%)	2338/6514 (35.9%)	1.02	0.90 to 1.16	0.43	0%	0.75
Trauma	3	12/98 (12.2%)	20/131 (15.3%)	0.78	0.35 to 1.72	0.14	48%	0.54
Witnessed cardiac arrest	2	343/1197 (28.7%)	1852/6503 (28.5%)	0.99	0.87 to 1.14	0.55	0%	0.93
Bystander CPR	3	782/1229 (63.6%)	4035/6556 (61.5%)	1.11	0.98 to 1.26	0.52	0%	0.11
Bystander AED	2	33/1192 (2.8%)	183/6496 (2.8%)	1.00	0.69 to 1.45	0.71	0%	0.99
The first recorded cardiac rhythm								
Shockable	4	71/1258 (5.6%)	383/6568 (5.8%)	0.98	0.75 to 1.27	0.85	0%	0.88
Non-shockable	4	1184/1258 (94.4%)	6183/6568 (94.2%)	1.00	0.77 to 1.30	0.60	0%	1.00
Airway management procedures								
ETI	2	40/57 (70.2%)	39/55 (70.9%)	1.30	0.11 to 15.53	0.03	80%	0.83
SADs	1	6/29 (20.7%)	8/18 (44.4%)	0.33	0.09 to 1.19	NA	NA	0.09
Outcomes								
ROSC	3	109/1221 (8.9%)	550/6514 (8.4%)	0.93	0.47 to 1.88	0.11	54%	0.85
Survival to hospital admission	4	128/1258 (10.2%)	590/6570 (9.0%)	0.81	0.45 to 1.44	0.09	54%	0.47
Survival to hospital discharge	2	148/1197 (12.4%)	872/6503 (13.4%)	0.62	0.22 to 1.72	0.05	73%	0.35
SHD with CPC 1–2	1	59/1160 (5.1%)	331/6443 (5.1%)	0.99	0.74 to 1.31	NA	NA	0.94

Legend: CI: confidence interval; CPC: Cerebral Performance Categories Scale; CPR: cardiopulmonary resuscitation; ETI: endotracheal intubation; MD: mean difference; NA: not applicable; OR: odd ratio; ROSC: return of spontaneous circulation; SAD: supraglottic airway device; SD: standard deviation; SHD: survival to hospital discharge.

## Data Availability

The data that support the findings of this study are available on request from the corresponding author (L.S.).
